# The Operative Treatment of Sternoclavicular Joint Dislocations in Adults: A Systematic Review

**DOI:** 10.7759/cureus.73229

**Published:** 2024-11-07

**Authors:** Maariya A Tariq, Christos G Dragonas, Joshua Nadimi, Lina Abbakr, Dimitra Leivadiotou

**Affiliations:** 1 Trauma and Orthopedics, The Princess Alexandra Hospital NHS Trust, Harlow, GBR; 2 Trauma and Orthopedics, Royal London Hospital, London, GBR

**Keywords:** american shoulder and elbow surgeons score (ases), operative treatment, range of motion (rom), sternoclavicular joint dislocation, sternoclavicular joint injury, sternoclavicular joint plate, sternoclavicular joint (scj)

## Abstract

This systematic review aimed to assess the literature on the treatment modalities used for acute sternoclavicular joint (SCJ) dislocation injuries. We aimed to review the clinical outcomes following these modalities by assessing functional scores, range of motion (ROM), patient satisfaction, complications, and revisions. A thorough literature search was conducted on four databases (Cochrane Library, Embase, MEDLINE, and Google Scholar) for studies published from January 2000 to May 2024 that focused on surgical treatment of sternoclavicular joint dislocation in patients over the age of 18 years. Of 1509 studies identified, 16 met our inclusion criteria and were selected as eligible. The studies included a total of 245 patients with an average age of 42.7 years ranging from 15 to 75 years. A total of 247 acute SCJ dislocations were treated with an average follow-up period of 45.7 months. Our research demonstrated that operative treatment for both anterior and posterior SCJ dislocations is beneficial and has been shown to improve functional outcomes and patient satisfaction, with few complications and a low revision rate. There is a wide range of implants and devices available, and detailed diagnostics of injury type and patient demographics would help to aid in the selection of an optimal device. In order to have comparable data between these interventions, further research, including randomized controlled trials and comparable studies, needs to be conducted. This, in turn, would contribute to more specific guidelines in the future for the treatment of these injuries.

## Introduction and background

Injury and dislocation of the sternoclavicular joint (SCJ) is relatively uncommon; however, it is a significant injury affecting the quality of life of both young and older patients [1-3]. They typically present acutely from high-energy trauma, although chronic instability can be attributed to congenital collagen disorders, such as Ehlers-Danlos syndrome [4-6]. In dislocation injuries, the direction of the clavicle subluxation relative to the sternum defines the direction of instability, making it anterior or posterior [1,4,7].

Anterior dislocation is more common and is usually treated with a conservative approach initially, partially due to the lack of critical anatomy anterior to the joint [1,4,7]. Surgical intervention is sometimes indicated when the conservative approach fails to maintain stability or function [4,8]. Despite this consensus, multiple studies advocate for surgical reduction as an initial intervention for the reduction of SCJ dislocations as they report good functional outcomes and patient satisfaction [4,9].

Posterior dislocations pose a greater risk due to the hilar and mediastinal structures lying inferior to the SCJ [2,3]. These injuries therefore require urgent surgical intervention to reduce and stabilize the joint to minimize the chance of further dislocation [2,3,10]. Surgical stabilization can be achieved with various methods, which include locking plate fixation, hook plates, SCJ-specific plate systems, or tendinous grafts; however, the low incidence of these injuries requiring surgical stabilization results in a lack of supporting research into outcomes, and thus, definitive guidelines for management [4,10].

Diagnosis and appropriate management are paramount to preserving function, improving pain, reducing the risk of neurovascular complications, and obtaining optimal patient satisfaction [11,12]. To date, there is a notable lack of conclusive evidence regarding the optimal management of these injuries. This systematic review aimed to analyze the literature surrounding the management of acute SCJ dislocations, evaluate outcomes, and identify optimal methods of treatment. We sought to achieve this by utilizing and comparing different outcome measures of functionality and/or patient satisfaction. For this review, we have chosen to focus on acute SCJ injuries in the adult population [4].

## Review

Materials and methods

This systematic review was designed and executed according to the Preferred Reporting Items for Systematic Reviews and Meta-Analyses (PRISMA) guidelines and protocols. We established the eligibility criteria prior to commencing the search. The study was registered on the Prospective Register of Systematic Reviews (PROSPERO).

Search Strategy and Study Selection

The protocol for this study was based on a modified variation of the Population, Intervention, Control, and Outcomes (PICO) model, adjusted for observational studies. A detailed literature search was performed on four databases (Cochrane Library, Embase, MEDLINE, and Google Scholar) for studies that were published from January 2000 to May 2024 and focused on surgical treatment of sternoclavicular joint dislocation in patients over the age of 18 years.

The search strategy encompassed Medical Subject Headings (MeSH) and free-text terms that delineate the exposure variable as well as the population of interest. The following search term was utilized: Sternoclavicular Joint AND (dislocation OR Injury). Only studies that were published in English or had English as their primary language were included. Following duplicate removal, two independent reviewers (MT, CD) assessed the titles, abstracts, and full texts of the selected studies. Any differences or discrepancies between the two reviewers were resolved by a third reviewer prior to data extraction.

Eligibility Criteria

The following inclusion criteria were used: (1) studies that examined patients surgically treated for sternoclavicular joint (SCJ) dislocation, (2) a patient population aged over 18 years, (3) patients treated for either anterior or posterior dislocation, and (4) minimum follow-up time of 12 months. We excluded studies that included (1) patients aged less than 18 years, (2) focused solely on conservative management of SCJ dislocations, (3) included less than five patients, and (4) congenital dislocations or pathological fractures. We included randomized controlled trials, cohort studies, case series, and case-control studies. Biomechanical studies, technical notes, expert opinions, review articles, letters to the editor, meta-analyses, conference abstracts, and case reports were excluded from the analysis.

The primary outcome of this review was defined as the American Shoulder and Elbow Surgeons Score (ASES). Secondary outcomes included Constant Score/Constant Shoulder Score/Constant-Murley Score (CS/CSS/CMS); Disabilities of the Arm, Shoulder and Hand (DASH) score, visual analog scale (VAS) score, University of California Los Angeles (UCLA) Shoulder score, range of motion (ROM), complications, and revisions. We also extracted complication rates and further subclassified them depending on their type. We aimed to identify similar clinical outcomes reported in the selected studies that could aid a comparison.

Data Extraction

Following study selection, data extraction was conducted on the studies which included the following variables: (1) first author’s name, (2) publication year, (3) study design, (4) sample size measured in number of patients and in number of operated joints, (5) average age in the patient population, (6) average follow-up duration in months, (7) type of procedure, (8) number of operated joints, (9) functional outcome measures including ASES, CSS, DASH score, VAS score, and (10) revision rate, (11) overall complications.

Quality/Risk of Bias Assessment of the Included Studies

The methodological index for non-randomized studies (MINORS) criteria were used to carry out a quality assessment of the included studies. Each of the 12 items in the MINORS criteria is scored between zero (0) and two (2), with a maximum score of 12 for non-comparative and 24 for comparative studies.

Results

**Figure 1:**
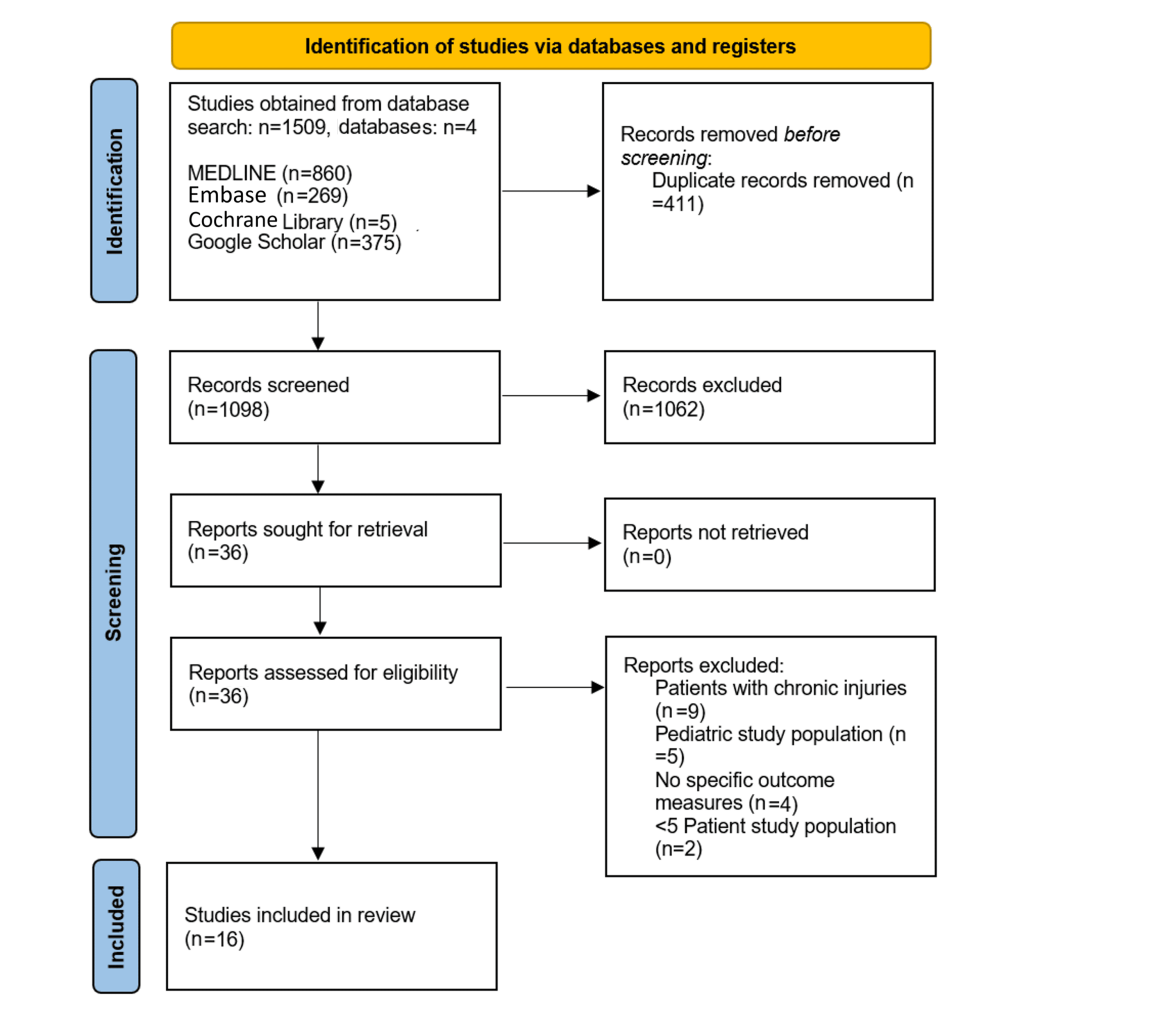
Preferred Reporting Items for Systematic Reviews and Meta-Analyses flowchart.

Our initial literature research included 1509 studies, from which 411 duplicates were removed. A further 1062 papers were excluded based on the title and abstract leaving 36 studies. Following a full-text review, 16 of these studies met our inclusion criteria. The study selection process is outlined in the flowchart in Figure 1.

The studies included a total of 245 patients with a mean age of 42.7 years (range: 15-75 years). A total of 247 acute SCJ dislocations were treated with a mean follow-up of 45.7 months. Four studies solely focused on anterior SCJ dislocations and five focused on posterior dislocations. The remaining nine studies either did not specify the nature of the dislocation or did not make a distinction between the two when presenting their outcome results. The full details of the included studies are summarized in Table 1.

**Table 1:** Studies included in the review. ACJHP: acromioclavicular joint hook plate; LP: locking plate; SCJ: sternoclavicular joint; ASES: American Shoulder and Elbow Surgeons Score; DASH: Disabilities of the Arm, Shoulder and Hand; ROM: range of motion; CS: Constant score/Constant-Murley score; VAS: visual analog scale; UCLA: University of California Los Angeles

Study	Year	Joints	Patients	Follow-up	Intervention	Anterior dislocations	Posterior dislocations	Age (years)	Outcome scores used
Ibrahim et al. [13]	2023	10	10	12 (range: 9-18) months	Non-absorbable tape sutures	10	0	40.1 (range: 18-52)	ASES, DASH
Tytherleigh-Strong et al. [14]	2018	6	6	28.2 (range: 24-35) months	Suture repair with internal brace	6	0	28.3 (range: 18-49)	QuickDASH
Qu et al. [15]	2019	12	10	16.9 (range: 10-24) months	ACJHP	12	0	43.6 (range: 28-64)	ASES
Qu et al. [16]	2022	17	17	14.4 (range: 3-28) months	ACJHP or LP	17	0	49.5	Clinical ROM assessment only
Xin et al. [17]	2023	11	11	18.00±3.74 months	Hook plate	0	11	54.91±13.58	CS, Rockwood
Tytherleigh-Strong et al. [18]	2022	17	17	94.5 (range: 25-155) months	Hamstring tendon autograft	0	17	30.8 (range: 18-52)	CS, QuickDASH, Rockwood
Groh et al. [9]	2011	21	21	5 (range: 2-16) years	Conservative and suture repair	0	21	30 (range: 24-54)	UCLA shoulder score
Zhang et al. [19]	2022	16	16	15.6 (range: 9-12) months	LP or an inverted hook plate	Unspecified	Unspecified	42.00±10.00 (range: 25-56)	ASES
Feng et al. [20]	2023	34	34	30.7±26.5 months	LP and trans-articular clavicle hook plate	Unspecified	Unspecified	50.0±14.8 (range: 18-75)	DASH, VAS, CS
Ao et al. [21]	2018	5	5	14 (range: 11-16) months	LP	3	2	36.2 (range: 29-43)	CS, DASH
Zheng et al. [22]	2018	12	12	22.7 (range: 12-30), 3, 6, 12 months	T plate	Unspecified	Unspecified	44.1±9.1 (range: 25-59)	VAS, Rockwood
Titchener et al. [23]	2019	8	8	30.5 (range: 18- 39) months	90 Degrees contoured plate	Unspecified	Unspecified	34.3 (range: 15-59)	QuickDASH
Franck et al. [24]	2003	10	10	1 year or more	Balser plate	6	3 (1 multidirectional)	33.4 (range: 17-61)	CS, DASH
Feng et al. [25]	2020	17	17	20.1±7.9 months	Balser plate	11	1	45.6±15.5 (range: 15-17)	DASH, VAS, CS
Wang et al. [26]	2021	22	22	94.8 months	SCJ specific plate	18	4	45.6 (range: 17-66)	VAS, ASES
Zhu et al. [27]	2022	31	31	98.5 (range: 13-171) months	SCJ specific plate	Unspecified	Unspecified	47.5 (range: 29-72)	ASES, VAS

Methodological Items for Non-randomized Studies (MINORS) Score

The mean MINORS score was 9.625 out of 16 for non-comparative studies (range of scores: 8-12). Points were lost in all studies for retrospective study design and prospective calculation of sample size. All studies lacked an unbiased assessment of the study endpoint. Four studies lost two points due to patients being lost to follow-up (Table 2).

**Table 2:** Methodological Items for Non-randomized Studies (MINORS) score. The items are scored 0 (not reported), 1 (reported but inadequate), or 2 (reported and adequate). The global ideal score is 16 for non-comparative studies and 24 for comparative studies.

MINORS	Ibrahim et al. [13]	Tytherleigh-Strong et al. [14]	Qu et al. [15]	Qu et al. [16]	Xin et al. [17]	Tytherleigh-Strong et al. [18]	Groh et al. [9]	Zhang et al. [19]	Feng et al. [20]	Ao et al. [21]	Zheng et al. [22]	Titchener et al. [23]	Franck et al. [24]	Feng et al. [25]	Wang et al. [26]	Zhu et al. [27]
1. A clearly stated aim	2	2	2	2	2	2	2	2	2	2	2	2	2	2	2	2
2. Inclusion of consecutive patients	2	2	2	2	2	2	2	2	2	2	2	2	2	2	2	2
3. Prospective collection of data	2	0	0	0	0	0	0	0	0	0	0	0	0	0	0	0
4. Endpoints appropriate to the aim of the study	2	2	2	2	2	2	2	2	2	2	2	2	2	2	2	2
5. Unbiased assessment of the study endpoint	0	0	0	0	0	0	0	0	0	0	0	0	0	0	0	0
6. Follow-up period appropriate to the aim of the study	2	2	2	2	2	2	2	2	2	2	2	2	2	2	2	2
7. Loss to follow-up <5%	2	2	2	2	2	0	0	2	2	2	2	2	0	2	2	0
8. Prospective calculation of the study size	0	0	0	0	0	0	0	0	0	0	0	0	0	0	0	0
9. An adequate control group	N/A	N/A	N/A	N/A	N/A	N/A	N/A	N/A	N/A	N/A	N/A	N/A	N/A	N/A	N/A	N/A
10. Contemporary groups	N/A	N/A	N/A	N/A	N/A	N/A	N/A	N/A	N/A	N/A	N/A	N/A	N/A	N/A	N/A	N/A
11. Baseline equivalence of groups	N/A	N/A	N/A	N/A	N/A	N/A	N/A	N/A	N/A	N/A	N/A	N/A	N/A	N/A	N/A	N/A
12. Adequate statistical analysis	N/A	N/A	N/A	N/A	N/A	N/A	N/A	N/A	N/A	N/A	N/A	N/A	N/A	N/A	N/A	N/A
Total (out of 24)	12	10	10	10	10	8	8	10	10	10	10	10	8	10	10	8

Anterior Dislocations

Four studies that focused on anterior dislocations used the treatment modalities of either hook plates, suture tapes, or suture taping with brace reinforcement [13-16]. The first of the four was conducted in 2023 by Ibrahim et al., which included 10 patients with an average age of 40.1 years (range: 18-52 years) and a mean follow-up of 12 months [13]. They conducted open reconstruction using high-strength non-absorbable tape sutures [13]. Their outcome measures included the use of the American Shoulder and Elbow Surgeons Score (ASES), which ranges from 0 to 100 (0 is the lowest level of function, and 100 is the highest). The ASES showed a significant improvement in mean physical function by 24.59 (pre-operative score 68.11±3.72, post-operative score 92.70±3.54) (p<0.00001) [13]. DASH score demonstrated significant improvement from 68.92±4.38 to 5.16±2.91 post-operatively [13]. All patients regained their pre-injury activity level, with one case of subluxation that was reported as negligible and did not impact joint function; therefore, no revisions were made [13].

Tytherleigh-Strong et al. presented their case series analysis on the use of anterior capsule suture repair and augmentation with internal tape bracing for traumatic anterior SCJ dislocation [14]. The study evaluated six patients with a mean age of 28.3 years (range: 18-49 years) [14]. Median follow-up was 28.2 months (range: 24-35 months) [14]. The internal brace involves braided, ultra-high-molecular weight polyester suture tape and knotless bone anchors to reinforce ligament strength as a secondary stabilizer following capsular suture repair [14]. At the most recent follow-up, no patients experienced further dislocations, and all considered their joints to be stable, returning to sporting activity within 12 months [14]. Clinically, no difference with regard to instability or range of motion (protraction, retraction, elevation, internal and external rotation) between the operated and uninjured SCJs was noted [14]. A short version of the Disabilities of the Arm, Shoulder and Hand (QuickDASH) showed a mean score of 2.3 (range: 0-4.5), and no complications were reported [14].

Two studies utilized acromioclavicular joint hook plates (ACJHP) as their treatment modality. The first study by Qu et al., which was published in 2019, included 12 acute anterior SCJ dislocations in 10 patients with a mean age of 43.6 years (range: 28-64 years) and an average follow-up period of 16.9 months [15]. They inserted the ACJHP into the dorsal osteal face of the sternal manubrium, and a lever effect was taken to press the medial end of clavicle down [15]. Ligament repair was carried out with absorbable sutures [15]. ASES showed an improvement from a pre-operative average score of 83.5 to 94.8 [15]. They also utilized the following post-operative measurements of range of motion: mean abduction angle of the glenohumeral joint being 164.3 (range: 153-172), posterior extension was 39.9 (range: 30-44), forward elevation was 147 (range: 135-165), and horizontal extension was 24.5 (range: 21-29) [15]. However, they did describe difficulties with the plate not being in line with the joint, and on this basis designed a new hook plate to conform to the SCJ structure to trial in future studies [15].

The second study by Qu et al., published in 2022, involved 17 patients with an average age of 49.5 years and a mean follow-up period of 14.4 months (range: 3-28 months) [16]. It assessed the use of an acromioclavicular joint hook plate (AJHP) compared to a locking plate (LP) by analyzing the range of motion [16]. Patients treated with AJHP fixation demonstrated better post-operative abduction (164.2857±8.86405 vs. 146.8182±11.46140, p=0.004), posterior extension (45.7143±5.34522 vs. 32.7273±8.47456, p=0.002), and external rotation (71.4286±10.29332 vs. 57.7273±6.84238, p=0.004) compared to patients treated with LP [16]. One patient in the LP group had a pneumothorax post-operatively, but also underwent same-side rib fracture internal fixation in the same operation [16]. This study also provides support for the use of the AJHP in addition to the first study published in 2019 [15].

Posterior Dislocations

Five studies that met our inclusion criteria included solely posterior SCJ dislocations. Xin et al., in 2023, retrospectively assessed 11 patients with posterior SCJ dislocation and an average age of 54.91 years (range: 33-71 years) with a mean follow-up period of 18 months [17]. The patients underwent surgical fixation with a thoracic locking hook plate and all plates were removed 12-18 months post-operatively [17]. This study used the Constant-Murley score and the Rockwood score, both encompassing range of motion (ROM) and pain [17]. The mean Constant-Murley score was 93.64±9.01 at 12 months post-operatively [17]. The VAS pain score was 14.09±2.02 while the ROM score was 36.36±4.80 points [17]. The Rockwood score was out of a maximum of 15; a total score <7 was poor, 7-9 was fair, 10-12 was good, and 13-15 was excellent [17]. The Rockwood score was an average of 13.36±1.86, pain was 2.82±0.41, ROM was 2.27±0.65, and the subjective result was 2.73±0.65, of which nine cases were excellent, one case was good, and one was fair [17].

In another study by Tytherleigh-Strong et al., published in 2022, 17 patients with an average age of 30.8 years (range: 18-52 years) were treated with hamstring tendon autograft reconstruction after acute posterior SCJ dislocation [18]. The mean follow-up was 94.5 months (range: 25-155 months) [18]. A figure-of-eight reconstruction was performed using a hamstring tendon autograft [18]. SCJ function was assessed using the following scores: QuickDASH, Rockwood SCJ, and modified Constant-Murley score [18]. At the final follow-up, the mean scores were as follows: QuickDASH=4.3 (range: 0-20.4); Rockwood=13.9 (range: 12-15); modified constant=94.4 (range: 71-100) [18]. When asked, all patients considered their SCJs stable, and 14 considered that the injured SCJ was now the same as the other side post-operatively [18]. Two patients failed to return to sports due to other factors, such as lack of confidence, age, and other injuries [18]. Two patients developed superficial wound infections, both of which settled with a short course of oral antibiotics [18]. One patient had symptoms of post-traumatic arthritis in the SCJ three years after surgery and had successful arthroscopic excision of the end of the medial clavicle [18].

Groh et al. treated patients operatively following a failed conservative approach [9]. Twenty-one patients with a mean age of 30 years (range: 24-54 years) and an average follow-up of five years (range: 2-16 years) were assessed [9]. All patients had an initial trial of closed reduction, via an abduction traction technique which successfully retained stability in eight patients [9]. These eight patients were then grouped into the first group with an average age of 39 years (range: 25-54 years) [9]. The second group involved 13 patients with a mean age of 36 years (range: 24-45 years) who had surgical intervention after a failed closed reduction [9]. The procedure involved the resection of 1.5-2.0 cm of the medial clavicle and the use of Cottony Dacron sutures to pass around the remaining medial clavicle, periosteum, and ligaments to stabilize and reduce the joint [9]. This was then further stabilized with several non-absorbable sutures [9]. Results were evaluated with respect to pain, range of motion, strength, function, and patient satisfaction according to the University of California, Los Angeles rating scale. Scores range from 0 to 35 with a score of 0 indicating worse shoulder function and 35 indicating better shoulder function [9].

When analyzing group one, closed reduction/conservative management was more likely to be successful (p<0.05) if carried out within 10 days of injury [9]. The UCLA shoulder scores in group one had a mean of 31 points (range: 29-35) [9]. Of the eight shoulders, three were classified as an excellent result and four were classified as a good result [9]. Four patients reported normal function with all activities, but the other four noted a slight restriction [9]. All patients showed >150 degrees of active elevation and were satisfied with the results [9].

In group two, an excellent result was achieved in five of the 13 operatively treated shoulders, a good result in six, and a fair result in two [9]. The UCLA shoulder scores for group two patients ranged from 27 to 35 points (mean: 32 points) [9]. All patients had more than 150 degrees of active elevation [9]. Seven patients reported normal function with all activities of daily living, work, and sports while three patients had negligible restriction while using the operated limb above the shoulder level for different sports or work activities. Three patients had more than slight restriction when doing this, but were not restricted when the limb was being used below shoulder level [9].

Non-Specific Dislocations

Nine studies analyzed different treatment modalities for SCJ dislocations; however, they did not specify individual measurement outcomes relative to anterior and posterior dislocations specifically. Zhang et al. focused on a SCJ hook plate in 16 patients with a mean age of 42 years (range: 25-56 years) and a follow-up period between 9 and 12 months [19]. In anterior dislocation, the clavicle was repositioned posteriorly and then the plate was fixed to the clavicle with screws [19]. In posterior dislocations, after reduction, a screw spacer and screw cap were added to the threads at the hook end to prevent recurrence of dislocation [19]. The average ASES score significantly improved from 49±4 (pre-operative score) to 91±3 (12 months follow-up) (p<0.001)[19]. Internal fixation failure and fracture non-union complications were seen in two patients [19].

Another study by Feng et al. also looked at the use of inverted hook plates in addition to extra-articular locking plates [20]. It involved 34 patients, 16 of which were treated using a trans-articular hook plate and 18 treated with the locking plate [20]. The average patient age was 50 years (range: 18-75 years) with an average follow-up period of 30.7 months [20]. Thirty-three patients (97.1%) achieved bone healing and had a mean Constant-Murley score of 90.9±11.0 (range: 50-100) [20]. Shoulder function was excellent in 29 patients, good in three, fair in one, and poor in one patient [20]. Mean shoulder forward flexion was calculated as 157.9±22.0 (range: 90-175), the mean DASH was 6.0±6.6 (range: 0-33), and the mean VAS was 0.4±1.1 (range: 0-5) [20]. Two patients had hook migration and went on to have revision surgery and had no further migration during follow-up [20]. Thirty-two returned to their daily activities prior to the injury, and 15 had implant removal after the bone union had occurred [20]. The study reports both plates to be an adequate option but no comparative statistical analysis was done comparing the two methods [20].

Locking plates were also used in a case series by Ao et al., published in 2018, which involved five patients [21]. Patients had a mean age of 36.2 years and were followed up after an average of 14 months [21]. All patients had secondary operations for plate removal six months post-operatively [21]. The final follow-up demonstrated a mean Constant Shoulder Score and DASH score of 89.5 (range: 78-98) and 9.0 (range: 4-16), respectively [21]. Four out of five patients were satisfied with the outcomes [21]. All patients were able to return to their previous activities [21].

A study by Zheng et al., published in 2018, also involved locking plates but the study is mainly focused on T plate fixation combined with repairing the anterior sternoclavicular ligament with sutures for patient with SCJ dislocation combined with proximal fracture [22]. Twelve patients with a mean age of 44.1 years were studied and followed up three, six, and 12 months after the procedure, with the most recent follow-up occurring an average of 22.7 months post-procedure [22]. According to the Rockwood SCJ scoring system, the average score was 7.7±0.75 pre-operatively and 13.3±0.49 at 12 months follow-up [22]. Meanwhile, VAS pain score was 7.9±1.15 pre-operatively and 2.1±1.07 at 12 months follow-up with the difference being statistically significant (p<0.05) [22]. One patient had a re-dislocation after implant removal [22].

In another study, Titchener et al. looked at eight patients with an average age of 31.3 years who had sustained an acute, displaced fracture of the medial end of the clavicle and had undergone operative fixation using an inverted distal clavicle plate contoured through 90 degrees with an average follow-up of 30.5 months [23]. The 11-item version of the DASH score was used and showed an average of 0.6 (range: 0-2.3) points [23]. All patients had returned to their pre-injury level of activity and reported the same range of motion in the injured and uninjured shoulder [23]. Two patients felt that their metalwork was slightly prominent but chose not to have it removed [23]. All patients said that they would be happy to undergo the procedure again if needed on the contralateral side [23].

Two studies focused on the use of Balser Plates in their patient population. The first study by Franch et al., in 2003, looked at 10 patients ranging from 17 to 61 years, six of which had anterior dislocations, three being posterior, and one classified as multidirectional instability [24]. Patients had temporary fixation with K wires then the hook of the Balser plate was shaped to match the contour of the manubrium and inserted [24]. The mean DASH score was 8.4±1.4 while the mean Constant-Murley score was 90.2±6.6, a figure which may only be interpreted as a trend because of the low number of patients [24]. No case of re-dislocation was observed [24]. There was one case of a post-operative seroma that was surgically drained after two months [24].

The second study by Feng et al. using Balser plates was published in 2020 [25]. It focused on 17 patients with an average age of 45.6 and a mean follow-up of 20.1 months [25]. Outcome measures showed a mean DASH score of 5.2±5.2 (range: 0.0-18.3) and a mean CS score of 93.7±7.9 (range: 72-100), with 15 cases being excellent, and two cases being good [25]. The mean VAS score was 1.1±1.4 (range: 2-7) at the final follow-up, showing significant improvement compared with the VAS score pre-operatively (4.9±1.3) (p<0.05) [25]. Mean shoulder forward flexion was measured at 162.9±8.1 (range: 50-180) [25]. Fifteen patients returned to their pre-injury daily activities, and all were satisfied with their treatment outcome [25]. There was one case of a wound hematoma three days post-operatively, which was debrided, and one patient underwent revision reconstruction for recurrent instability due to hook migration seven days post-operatively [25].

The final two studies we looked at focused on a SCJ-specific plate. The first one by Wang et al., published in 2021, focused on long-term results [26]. They looked at 22 patients, 18 with anterior dislocations and four with posterior, and an average age of 45.6 years (range: 17-66 years) [26]. An aluminum template was first molded according to the reduced SCJ, and then an anatomical plate was contoured according to the aluminum template [26]. This was fixed with bi-cortical screws [26]. In cases of posterior dislocation or multidirectional instability, nuts and washers were implemented [26]. Patients were assessed after an average follow-up period of 94.8 months [26]. All implants were routinely removed 6-12 months post-operatively [26]. The ASES improved significantly from 37.9±10.1 to 90.8±7.8 at implant removal (p<0.001) and to 86.7±8.6 at the final follow-up (p<0.001) [26]. The VAS significantly improved from 7.1±1.3 to 0.9±1.0 at implant removal (p<0.001) and to 1.2±1.1 at the latest follow-up (p<0.001) [26]. Abduction significantly improved from 83.6±8.8 degrees to 162.5±18.1 at implant removal (p<0.001) and to 157.8±15.3 at final follow-up (p<0.001) [26]. Forward elevation also improved from 110.5±16.5 to 149.6±17.2 (p<0.001) and to 144.7±18.1 at the final follow-up (p<0.001) [26]. Sternal osteolysis occurred around the hook in one patient with osteoporosis but the SCJ remained stable, and the implant was removed without complaints at 13 months follow-up [26].

An SCJ-specific plate was also used by Zhu et al. for 31 patients with SCJ dislocations and associated medial-end clavicle fractures [27]. The average age was 47.5 years (range: 29-72 years) with a follow-up ranging from 13 to 171 months [27]. The mean ASES score increased significantly from 34.3±7.8 pre-operatively to 90.2±4.9 at the final follow-up (p<0.001) [27]. The mean VAS score significantly decreased from 6.8±1.0 to 0.9±0.8 at the final follow-up (p<0.001) [27]. Mean shoulder abduction significantly increased from 72.1±6.6 to 169.5±8.5 at the final follow-up (p<0.001) and the mean shoulder forward elevation significantly increased from 97.1±11.0 to 163.1±11.5 at the final follow-up (p<0.001) [27]. All patients had their implants removed after a mean of 10.7 months (range: 8-14) post-operatively [27]. This study also observed an enlargement of the hole in the sternum during the removal of the implant [27]. No other complications were noted [27].

Discussion

The infrequent occurrence of SCJ dislocations has resulted in a paucity of literature and evidence base regarding management. This systematic review has demonstrated that numerous different surgical techniques are proven to be successful in the treatment of SCJ dislocations.

The use of acromioclavicular joint hook plates (ACJHP) has been analyzed in the literature and their successful outcomes have been demonstrated in multiple studies [15-17,19,20]. With respect to anterior dislocations specifically, ACJHP fixation has shown promising results. One study demonstrates better outcomes in terms of shoulder range of motion compared to standard locking plate fixation [16]. Various studies have therefore opted to examine ACJHP further, as it is adapted to be more specific for the SCJ [16]. Studies by Wang et al. and Zhu et al. examine outcomes associated with SCJ-specific plates [26,27]. Both studies demonstrated successful outcomes and significant improvement in range of motion, ASES, and VAS scores after an SCJ-specific molded plate was used [26,27]. These studies convey the benefits of hook plate use specifically designed for post-traumatic SCJ fixation and enhance the support for further research into this fixation modality and how they may be optimized for this acute indication.

An alternative hook plate design, the Balser plate, has demonstrated success in two of the studies included in our research [24,25]. Both papers reported a significant improvement in DASH and Constant-Murley scores, albeit in a small sample size [24,25]. One of these papers demonstrated 100% patient satisfaction with 88% of the patients returning to similar activity levels as before injury [25]. Feng et al. concluded that the Balser plate is a good intervention for SCJ dislocations; however, complications were encountered in the study population which included seroma, hematoma, or impingement due to hook migration [24,25]. Furthermore, it was noted that the plate's size and shape are large compared to the small SCJ, which can pose technical difficulties, result in the prominence of metalwork, and contribute to a less-than-optimal outcome [24]. This advocates the use of SCJ-specific plates, where fewer complications are reported in the literature; however, further research is required into the use of specific SCJ molded plates to confirm this.

The majority of the literature investigates outcomes in relation to locking plate use in anterior SCJ dislocations. However, one particular study reported superior results with ACJHP use in acute SCJ injuries and demonstrated a better range of motion post-operatively [16,21]. Regarding posterior dislocations, thoracic locking hook plates have been shown to achieve an improvement in range of motion, pain, and quality of life [17]. Trans-articular locking plates have also been shown to be effective in the fixation of acute posterior dislocations. Patients regained their function and mobility post-operatively which was also reflected among patients with unspecified modes of SCJ dislocation [20]. There are also studies assessing the use of 90-degree contoured locking plates which have also shown to improve range of motion, return to activity, and patient satisfaction [23]. They demonstrate effectiveness in providing various fixation options, which supports the use of a plate specifically designed for the SCJ to enable stable fixation [23].

Overall, the literature extensively supports the effectiveness and versatility of locking plates [21,23]. However, numerous studies indicate that the ACJHP yields superior post-operative outcomes when compared to locking plates. These studies recommend conducting more comparative research, particularly against joint-specific and ACJHP interventions which have shown promising initial results [15,16,26,27]. Evidence provided by comparative studies of both plate systems would give a better understanding of the optimal intervention for acute SCJ dislocations.

This systematic review has found the use of suture tape fixation for both anterior and posterior SCJ dislocations to be effective [13,14]. Patients with anterior dislocations showed improved range of motion and returned to the previous level of activity when suture tape was used [13,14].

Based on our research, a single study compared conservative and surgical interventions with sutures for posterior SCJ dislocations [9]. The study concluded that both approaches are equally favorable, with successful outcomes and cases of mild pain and restriction [9]. All patients who underwent surgery had initially failed conservative treatment [9]. Therefore, it is recommended to first try conservative management to avoid unnecessary invasive procedures. However, further comparative research on conservative management and different surgical interventions would be beneficial.

This study demonstrates a wide range of fixation modalities to be effective in the management of acute SCJ dislocations. The use of ACJHP and SCJ-specific plates has shown success in terms of outcomes and patient satisfaction, and potential for further research into SCJ-specific molded plates [15,16,26,27].

Limitations

There is a paucity of high-level evidence in the literature, including randomized controlled trials investigating the use of different surgical techniques. Most studies are retrospective, hence limiting control of the study population and its variables.

Due to the variety of functional outcome scores used across all studies, it is challenging to compare studies directly. We minimized this by only using the scores which were comparable. It is also important to note that we did not conduct the evaluation for the risk of bias that inherently stems from the application of different surgical techniques in different demographic populations present in our included studies. Few studies have conducted statistical analysis to prove the significance of measurable outcomes, such as range of motion. Even fewer studies demonstrate a comparison between two or more methods. Finally, some of our studies lack a long-term follow-up which is required for the evaluation of the effectiveness of the studies’ interventions.

## Conclusions

Operative management of both anterior and posterior SCJ dislocations has been shown to improve functional outcomes, patient satisfaction, few complications, and a low revision rate. When planning the treatment of an acute SCJ dislocation, the surgeon must consider the type of dislocation, patient-specific factors, and a variety of fixation modalities. The multitude of available implants for the stabilization of such injuries opens up exciting future research prospects to enhance our understanding of how to manage these debilitating injuries.
